# Effects of Human Mesenchymal Stem Cells Isolated from Wharton's Jelly of the Umbilical Cord and Conditioned Media on Skeletal Muscle Regeneration Using a Myectomy Model

**DOI:** 10.1155/2014/376918

**Published:** 2014-10-14

**Authors:** T. Pereira, P. A. S. Armada-da Silva, I. Amorim, A. Rêma, A. R. Caseiro, A. Gärtner, M. Rodrigues, M. A. Lopes, P. J. Bártolo, J. D. Santos, A. L. Luís, A. C. Maurício

**Affiliations:** ^1^Departamento de Clínicas Veterinárias, Instituto de Ciências Biomédicas de Abel Salazar (ICBAS), Universidade do Porto (UP), Rua de Jorge Viterbo Ferreira, No. 228, 4050-313 Porto, Portugal; ^2^Centro de Estudos de Ciência Animal (CECA), Instituto de Ciências e Tecnologias Agrárias e Agro-Alimentares (ICETA), Rua D. Manuel II, Apartado 55142, 4051-401 Porto, Portugal; ^3^Faculdade de Motricidade Humana (FMH), Universidade de Lisboa (UL), Estrada da Costa, 1499-002 Cruz Quebrada—Dafundo, Portugal; ^4^CIPER-FMH: Centro Interdisciplinar de Estudo de Performance Humana, Faculdade de Motricidade Humana (FMH), Universidade de Lisboa (UL), Estrada da Costa, 1499-002 Cruz Quebrada, Dafundo, Portugal; ^5^Departamento de Patologia e de Imunologia Molecular, Instituto de Ciências Biomédicas de Abel Salazar (ICBAS), Universidade do Porto (UP), Rua de Jorge Viterbo Ferreira, No. 228, 4050-313 Porto, Portugal; ^6^Instituto Português de Patologia e Imunologia Molecular da Universidade do Porto (IPATIMUP), Rua Dr. Roberto Frias s/n, 4200-465 Porto, Portugal; ^7^CDRsp-Centro Para o Desenvolvimento Rápido e Sustentado de Produto, Instituto Politécnico de Leiria, Rua de Portugal, Zona Industrial, 2430-028 Marinha Grande, Portugal; ^8^CEMUC, Departamento de Engenharia Metalúrgica e Materiais, Faculdade de Engenharia, Universidade do Porto, Rua Dr. Roberto Frias, 4200-465 Porto, Portugal; ^9^UPVET, Instituto de Ciências Biomédicas de Abel Salazar (ICBAS), Universidade do Porto (UP), Rua de Jorge Viterbo Ferreira, No. 228, 4050-313 Porto, Portugal

## Abstract

Skeletal muscle has good regenerative capacity, but the extent of muscle injury and the developed fibrosis might prevent complete regeneration. The *in vivo* application of human mesenchymal stem cells (HMSCs) of the umbilical cord and the conditioned media (CM) where the HMSCs were cultured and expanded, associated with different vehicles to induce muscle regeneration, was evaluated in a rat myectomy model. Two commercially available vehicles and a spherical hydrogel developed by our research group were used. The treated groups obtained interesting results in terms of muscle regeneration, both in the histological and in the functional assessments. A less evident scar tissue, demonstrated by collagen type I quantification, was present in the muscles treated with HMSCs or their CM. In terms of the histological evaluation performed by ISO 10993-6 scoring, it was observed that HMSCs apparently have a long-term negative effect, since the groups treated with CM presented better scores. CM could be considered an alternative to the *in vivo* transplantation of these cells, as it can benefit from the local tissue response to secreted molecules with similar results in terms of muscular regeneration. Searching for an optimal vehicle might be the key point in the future of skeletal muscle tissue engineering.

## 1. Introduction

Incomplete regeneration after traumatic muscle injury with residual functional deficiencies is a common problem in orthopedics and traumatology [1]. Unlike minor traumas to the skeletal muscle, major losses of muscular tissue architecture render the wound site unable to support a normal regeneration process [2]. In these clinical situations, there is formation of scar tissue due to collagen type I synthesis by fibroblasts, which appears in the provisional fibrin matrix derived from the local blood clot. During regeneration, the cytokine-mediated induction of local satellite cells that previously lied dormant between the basal lamina and sarcolemma and that led to the formation of centronucleated myofibers (myotubes) is not enough to completely restore the area affected by the loss of muscular tissue [3].

Previous studies have developed new experimental models in which a defined portion of the muscle tissue is removed, creating a myectomy defect, or a laceration is produced (myotomy) within the muscle [2, 4]. Nonetheless, these models could not standardize the lesion (different defect sizes) since they were performed by manual dissection with a scalpel blade. So, our research group developed a novel experimental muscle injury of the* tibialis anterior* muscle of the rat by using a biopsy punch to create a standardized myectomy (surgical) lesion. This was part of a preliminary study in which different types of skeletal muscle lesions (chemical, mechanical, and surgical) were induced in order to determine the most appropriate for our goals [5]. Several studies have already been published based on models such as myotomy (laceration) or myectomy, which can mimic the loss of muscular tissue in severe lesions [2, 4, 6–8]. Compared to the chemical, ischemia, and mechanical lesions, the myectomy model has the advantage of permitting the implantation of mesenchymal stem cells (MSCs) together with a vehicle that can also be considered as a scaffold for these cells in the host environment, rather than injecting a cellular suspension in the core of the lesion. There is evidence both from animal studies and from clinical trials showing that cell therapy involving different types of stem cells application have promising results considering functional and morphologic regeneration of the muscle [2, 9–11]. Among other promising cellular systems that could substitute satellite cells and engraft in regenerating muscle are stem cells isolated from human umbilical cord blood or MSCs present in umbilical cord matrix, the so-called Wharton's jelly [12].

There are technical or/and ethical difficulties in obtaining enough and appropriate stem cells from the bone marrow or from embryos (obtained from assisted reproduction techniques or somatic nuclear transfer for therapeutic purposes), which have limited the application of these cellular therapies. MSCs from Wharton's jelly of the umbilical cord possess stem cell properties and are capable of differentiating into neurogenic, osteogenic, chondrogenic, adipogenic, and myogenic cells* in vitro* [13]. They may therefore prove to be a potential cellular system for cell therapy, including targets such as stromal tissue and muscle, by replacing the degenerated cells, by producing growth factors, or by modulating the inflammatory local response [14, 15]. MSCs have become one of the most interesting agents for Regenerative Medicine. Scientific and clinical evidence have demonstrated that MSCs have the ability to migrate to specific sites of injury or of tissue regeneration, where they modulate the immune and the inflammatory responses and mobilize intrinsic cell reservoirs through a series of distinct paracrine mechanisms [16]. In addition, these cells represent a noncontroversial source of primitive mesenchymal progenitor cells that can be isolated after birth, cryogenically stored, thawed, and expanded for therapeutic uses [13].

Nowadays, the comprehensive characterization of MSCs secretome is becoming particularly important, as the factors secreted by these cells seem to be the main effectors of their therapeutic action [17]. The hypothesis that the CM, where these cells grow and expand in culture (so-called conditioned media—CM), could be an appropriate therapeutic product rich in growth factors comparable to HMSCs local application, seemed to be a rational approach to our study.

Cell delivery requires a matrix in which cells are suspended [18]. Floseal, Tisseellyo, and a hydrogel [19, 20] were evaluated and compared in terms of biological integration into the skeletal muscle tissue after the standardized myectomy. The inflammatory reaction triggered by these vehicles was the main topic taken into account. Nevertheless, the role of these biomaterials as scaffolds for the HMSCs was also an important factor, since they could influence and promote a gradual release of cell and growth factors during the regenerative process.

Floseal (Baxter SA, Switzerland) is a hemostatic matrix that consists of the combination of a bovine-derived gelatin matrix and a human-derived thrombin component. The gelatin-thrombin matrix can be prepared immediately before use, as a highly viscous gel [21]. Both components of Floseal promote hemostasis. In addition to their individual actions, the gelatin matrix and the thrombin interact synergistically to facilitate the formation of a stable clot at the bleeding site. One important issue about Floseal is that, unlike other fibrin sealants like Tisseellyo, it does not work in the absence of blood, since it provides hemostasis by a different mechanism. Because the gelatin-thrombin matrix is hydrophilic, it adheres well to wet tissue in contrast with fibrin glue. The swelling gelatin particles restrict blood flow and also provide a mechanically stable matrix around which the fibrin clot can form. These effects have proven to be of great value in several surgical techniques like the ones in vascular surgery [22]. Reductions in hemorrhage in Floseal-treated women undergoing a myomectomy are also encouraging and provide evidence for the ability of gelatin-thrombin matrix to reduce blood loss when applied immediately and directly to a bleeding muscular tissue [21, 23]. Fibrin alone or in combination with other materials has been used as a biological scaffold for MSCs [2]. Fibrin glue, when diluted, can effectively support survival and proliferation of mesenchymal stem cells [18]. Tisseellyo (Baxter SA, Switzerland) is a fibrin sealant indicated for use as an adjunct to hemostasis in patients undergoing surgery when control of bleeding by conventional surgical techniques (such as suture, ligature, and cautery) is ineffective or impractical and is also effective in heparinized patients. The use of this mesenchymal stem cell/fibrin hybrid scaffold system in orthopedic reconstructive surgery seems to be a promising approach in some clinical situations [24].

Hydrogels, three-dimensional (3D) networks of hydrophilic polymers, are appealing for biological applications because of their high water content, high permeability, biocompatibility, and the ability to be placed into critical defects in a minimally invasive manner. In the present experimental work, a cerium nitrate cross-linked hydrogel composed of alginate and sodium hyaluronate was tested for cell encapsulation.

The hypothesis that the HMSCs from Wharton's jelly of the umbilical cord could have a crucial role in the restoration of the muscle architecture after injury was questioned. They could be important in muscle regeneration as they can interact with the host cell population in the remodeling of the extracellular matrix. Using the myectomy model, the local application of both the HMSCs isolated from Wharton's jelly and the CM was performed, associated with the three previously mentioned biocompatible vehicles, in order to evaluate the muscle regeneration enhancement at 15 days (d15) postsurgery. This was carried out by establishing three different groups (vehicle alone, vehicle* plus* MSCs, and vehicle* plus* CM) for each one of the proposed vehicles, in order to select the most appropriate biomaterial. Since fibrin glue presented the best scores in the d15 trial in terms of local reaction after implantation, it was elected as the vehicle used for the 35 days (d35) trial [25].

## 2. Materials and Methods

### 2.1. HMSCs Preparation before Transplantation

**Figure 1:**
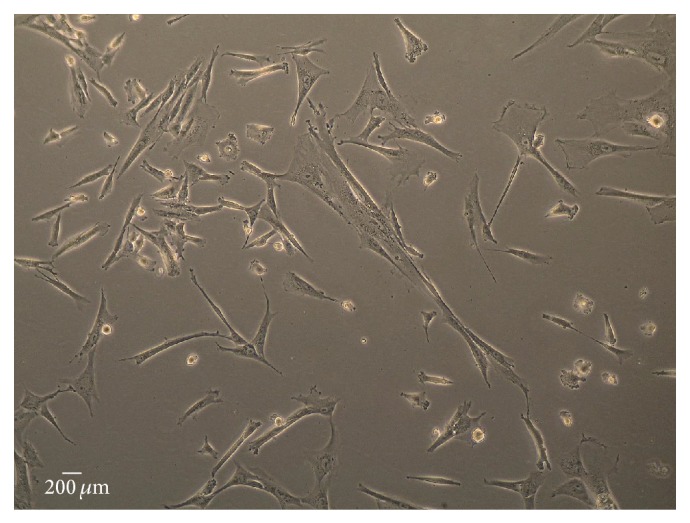
HMSCs from Wharton's jelly exhibiting a mesenchymal-like shape with a flat polygonal morphology after 4 days of* in vitro* culture (magnification: 100x).

HMSC from Wharton's jelly umbilical cord matrix were purchased from PromoCell GmbH (C-12971, lot number: 8082606.7). The HMSCs were cultured and maintained in a humidified atmosphere with 5% CO_2_ at 37°C. Mesenchymal Stem Cell Growth Medium PromoCell (C-28010), was replaced every 48 hours. At 80% confluence, cells were harvested with 0.25% trypsin with EDTA (GIBCO) and passed into a new flask for further expansion. HMSCs at a concentration of 10^4^ cells/cm^2^ were cultured exhibiting an 80% confluence after 4 days in culture medium. The phenotype of HMSCs was assessed by PromoCell assay. Rigid quality control tests were performed for each lot of PromoCell MSCs isolated from Wharton's jelly of UC. HMSCs were tested for cell morphology, adherence rate, and viability. Furthermore, each cell lot was characterized by flow cytometry analysis for a comprehensive panel of markers, such as platelet endothelial cell adhesion molecule-1 (PECAM-1, CD31), homing cell adhesion molecule (HCAM, CD44), CD45, and Endoglin (CD105). The HMSCs thawed and expanded in our laboratory exhibited a mesenchymal-like shape with a flat and polygonal morphology (Figure 1). The phenotype of the HMSCs expanded for the* in vivo* testing was confirmed in our laboratory by flow cytometry in order to certify that the HMSCs followed the International Society for Cellular Therapy (ISCT) criteria [26]. Detection was performed with the following antibodies and their respective isotypes (all from BioLegend unless stated otherwise): PE anti-human CD105 (eBioScience); APC anti-human CD73; PE anti-human CD90; PerCP/Cy5.5 anti-human CD45: FITC anti-human CD34; PerCP/Cy5.5 anti-human CD14; Pacific Blue anti-human CD19, and pacific-blue anti-human HLA-DR. Chromosome analysis on HMSCs from Wharton's jelly before* in vivo* application was carried out between passages 4 and 5. When 80% confluence was reached, culture medium was changed and supplemented with 4 *μ*g/mL Colcemid solution (stock solution, Cat. number 15212-012, Gibco, USA, New York). After 4 hours, cells were collected and suspended in 8 mL of 0.075 M KCl solution supplemented with bovine foetal serum. Then, the suspension was incubated in 37°C for 35 minutes (min). After centrifugation at 1500 rpm, the fixative methanol : glacial acetic acid (8 mL) was added at 6 : 1 and mixed together, and the cells were again centrifuged. After two rounds of fixation, two new rounds were performed with the fixative methanol : glacial acetic acid at 3 : 1. After the last centrifugation, the cell suspension was spread onto very well cleaned slides. Chromosome analysis was performed by one scorer on 20 Giemsa-stained metaphases. Each cell was scored for chromosome number. Routine chromosome G-banding analysis was also carried out for the determination of the karyotype. The karyotype of undifferentiated HMSCs was determined in order to certify the absence of neoplastic characteristics in these cells, as well as the chromosomal stability to the cell culture procedures before the* in vivo* application.

### 2.2. Conditioned Media (CM) Preparation before* In Vivo* Implantation

In order to obtain the CM, the HMSCs were thawed and plated out at a density of 4000 cells/cm^2^. They were maintained under the culture conditions described before, assessed continuously for viability, until obtaining a minimum of 80–90% confluence. The culture medium batch used in this study was checked for maintenance of multipotency, growth promoting activity, adherence rate, and typical morphology of the tested mesenchymal stem cells. This medium supports the expansion of HMSCs without inducing early senescence and differentiation. The culture medium was replaced every 48 hours and the CM was collected from the T75 flask for* in vivo* application only after reaching the desired cell confluence, in case the culture medium was in contact with the HMSCs for at least 48 hours. The CM was then concentrated in spin concentrators with a cut-off of 5 kDa (Agilent Technologies) as per manufacturers' recommendations.

### 2.3. Preparation of the Vehicles/Scaffolds for HMSCs Transplantation and Cell Viability

The gelatin matrix component of Floseal consists of cross-linked gelatin granules that swell 10 to 20% upon contact with blood or body fluids. This matrix, together with the human thrombin and the calcium chloride solution used for reconstitution of this latter component, are sterile and nonpyrogenic [21, 23].

**Figure 2:**
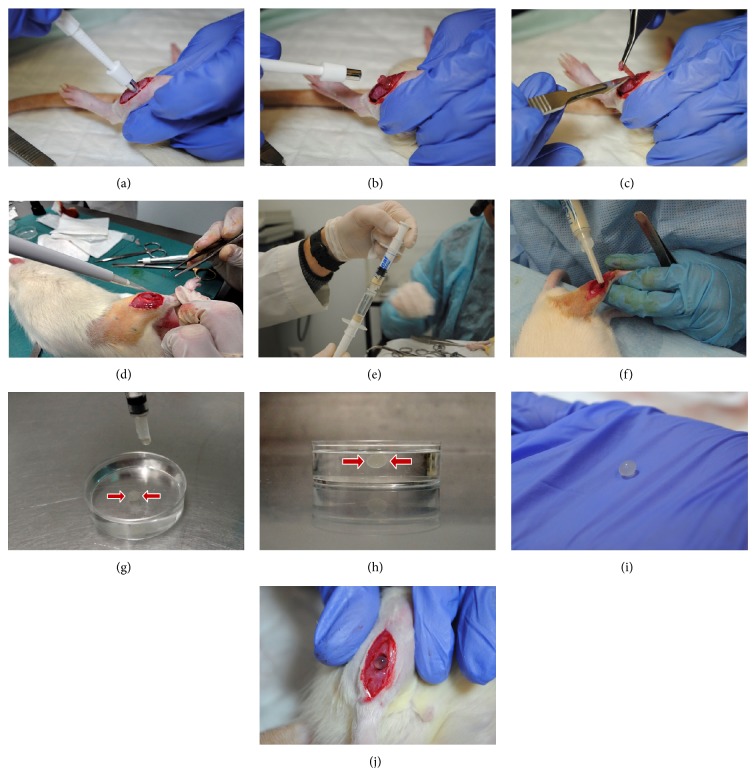
Biopsy punch for myectomy lesion creating a 5 mm Ø defect in the rat's* tibialis anterior* (TA) muscle (a–c). In the* MSCFibrin* group, the defect was filled with a cellular suspension containing 1 × 10^6^ HMSCs and 30 *μ*L of fibrin glue (Tisseellyo) (d). Preparation of Floseal: the gelatin matrix granules syringe is connected to the syringe containing the thrombin solution and the mixture is transferred back and forth between the syringes for a total of 10 passes (e). Application of Floseal in a 5 mm Ø myectomy defect (f). Preparation of the spherical hydrogel: the polymer solution containing alginate and sodium hyaluronate in a ratio of 1 : 1 (V/V) was inserted into an insulin syringe and a droplet was released into an excess of cerium nitrate solution 135 mM in order to obtain a cross-linked polymer sphere of approximately 60 *μ*L in volume (g–i). Application of the previously prepared spherical hydrogel in a 5 mm Ø myectomy defect (j).

Briefly, the preparation of Floseal starts by using a 5 mL syringe with needle attached provided in the thrombin component package; the 5 mL chloride solution is transferred to the vial containing the thrombin. The vial is gently swirled until the thrombin is dissolved. The dissolved thrombin is then aspirated from the small bowl into the syringe to the indicated mark (4 mL) and the gelatin matrix granules syringe is connected to the syringe containing the thrombin solution and the gelatin matrix—thrombin solution mixture is transferred back and forth between the syringes for a total of 10 passes (Figure 2(e)). The preparation time is approximately 1 minute, and, once completed, the mixture is usable for 2 hours (Figure 2(f)).

The fibrin matrix for HMSCs was prepared using a Tisseellyo kit following manufacturer's instructions. In summary, a 2 mL solution is prepared by adding 72–110 mg of fibrinogen, 10 IU factor XIII, and 3000 UIK of bovine aprotinin mixed with 500 IU of human thrombin and 40 *μ*M of calcium chloride.

In the groups containing cells and Floseal/Tisseellyo as vehicle, MSCs were resuspended in approximately 30 *μ*L of unconditioned culture media—Mesenchymal Stem Cell Growth Medium, PromoCell (C-28010), before combining to the prepared vehicle (Floseal and the fibrin matrix from Tisseellyo) for filling the 60 *μ*L skeletal muscle defect.

For preparation of the spherical hydrogel, the polymer solution was prepared by adding in a ratio of 1 : 1 (V/V) a sodium alginate aqueous solution 7% (m/V) to a sodium hyaluronate aqueous solution 0.5% (m/V), under magnetic stirring. Afterwards, the polymer solution was inserted into an insulin syringe and a droplet was released into an excess of cerium nitrate solution 135 mM in order to obtain a cross-linked polymer sphere of approximately 60 *μ*L of volume (approximately the same as the defect) (Figures 2(g)–2(j)). Cerium nitrate and sodium hyaluronate solutions were sterilized by microfiltration (0.22 *μ*m membrane) and sodium alginate powder was sterilized in an autoclave (120°C for 15 min) previous to the solution preparation. In the group in which MSCs were combined with hydrogel, cells were resuspended in the sodium alginate, sodium hyaluronate before cross-linking with cerium nitrate.

The spherical hydrogel not only was used as vehicle but its properties were also evaluated and optimized to find a suitable matrix for the cellular implants. This scaffold was previously developed and characterized from the physical-chemical and biological point of view [19, 20]. Intracellular free Ca^2+^ concentration ([Ca^2+^]_*i*_) was measured in Fura-2-loaded cells by using dual wavelength spectrofluorometry as previously described [27]. The measurements were performed on undifferentiated HMSCs after confluence was obtained cultured in the presence of Floseal, fibrin matrix from Tisseellyo, and spherical hydrogel in order to correlate the HMSCs survival capacity in the presence of the three used vehicles in the* in vivo* preclinical trials.

### 2.4. Surgical Procedure and HMSCs Transplantation

Sasco Sprague-Dawley male rats with 250–300 g were used. A standardized surgical lesion was performed using a 5 mm diameter biopsy punch blade, creating an approximately 60 *μ*L volume full thickness defect in the midbelly region of the* tibialis anterior* muscle (Figures 2(a)–2(c)). The defect was then completely filled with a cellular suspension containing 1 × 10^6^ HMSCs in 30 *μ*L of culture medium and 30 *μ*L of fibrin glue (Tisseellyo) containing fibrinogen and thrombin (*MSCFibrin* group) (Figure 2(d)). In another group (*ConditionedFibrin* group) 30 *μ*L of a CM, a concentrated media containing trophic factors from HMSCs in culture, was combined with 30 *μ*L of fibrinogen and thrombin.* Control* groups were also tested, with the 5 mm diameter myectomy lesion (*Control* group) and with the lesion combined with the addition of 30 *μ*L of fibrin (*Fibrin* group). Three more groups were also tested using an hemostatic matrix (Floseal) instead of the fibrin glue used previously (*Floseal*,* MSCFloseal*, and* ConditionedFloseal* groups) and three other groups using a hydrogel composed of hyaluronic acid, alginate, and cerium as a cross-linker (*Hydrogel*,* MSCHydrogel*, and* ConditionedHydrogel* groups). The surgeries were executed under general anesthesia with xylazine (1.25 mg/100 g BW ip) and ketamine (9 mg/100 g BW ip). The skin and the subcutaneous tissues were properly closed with a simple-interrupted suture of a 4/0 nonabsorbable filament (Synthofil). After 15 days, the animals were sacrificed and 6* tibialis anterior* muscles from each of the described groups (*N* = 6) were collected and fixed in 10% buffered formaldehyde for histological analysis. The* Control*,* Fibrin*, and* MSCFibrin* groups were also repeated in a different time point (d35) for the evaluation of the muscles after complete regeneration.

All the animal testing procedures were in conformity with the Directive 2010/63/EU of the European Parliament and with the approval of the Veterinary Authorities of Portugal in accordance with the European Communities Council Directive of November 1986 (86/609/EEC). Humane endpoints were followed in accordance with the OECD Guidance Document on the Recognition, Assessment, and Use of Clinical Signs as Humane Endpoints for Experimental Animals Used in Safety Evaluation (2000). Adequate measures were taken to minimize pain and discomfort taking into account human endpoints for animal suffering and distress. Animals were housed for two weeks before entering the experiment.

### 2.5. Histological Evaluation of the Local Effects

Tissue samples were fixed in 10% buffered formalin, routinely processed, dehydrated, and embedded in paraffin wax. Consecutive 3 *μ*m-sections were cut and stained with haematoxylin and eosin (HE).

The International Standard ISO-10993-6 [28] for biological evaluation of medical devices was employed for the assessment of the local effects after the implantation of the different biomaterials used as vehicles/scaffolds in this study [28]. The local effects were evaluated by a comparison of the tissue response caused by the tested implant to that caused by the* Control* and can be considered as nonirritant (<2.9 above* Control*), slight (3.0-8.9 above* Control*), moderate (9.0–15.0 above* Control*), and severe (>15.0 above* Control*) depending on the score obtained (semi-quantitative analysis). In this study, the* Control* group was obtained by performing the surgical procedure (myectomy) without any biomaterial or cell implantation. The effects of HMSCs and CM associated with fibrin glue in two distinct time points (d15 and d35) were also compared in order to observe the influence of these biological products in different stages of muscle regeneration. Each group was formed by 6* tibialis anterior* muscles and in each one of these, 18 fields were counted in a 400x magnification.

### 2.6. Collagen Quantification

Collagen content in the wound bed can be calculated by image analysis of Masson's Trichrome-stained histological images taken at a predefined image magnification [29]. A method for collagen quantification in skeletal muscle tissue was developed by our research group using the* ImageJ* ^*©*^ software (NIH) in order to compare the amount of fibrosis in the site of injury between the treated and untreated groups, throughout muscular regeneration.

An average of 5 Masson's Trichrome-stained histological images was captured from each sample at 20x magnification. To ensure that the analysis was executed well within the injury zone, the images were obtained from the muscular depth with the most observable collagen content [2]. These images were merged (by the* AutoStitch V2.2* software) to allow a complete visualization of the skeletal muscle area affected by the lesion. By using the threshold colour tool in the* ImageJ* ^*©*^ software (NIH), the areas stained in blue were selected and in that way we could indirectly calculate the fibrotic fraction of the skeletal muscle sample. This colour channel splitting and threshold analysis was performed for each image series collected using the same exposure, brightness, and white balance settings. The ratio of blue pixels above the threshold to total pixels in the image is used to calculate the percentage of fibrosis in each sample [30]. These values allowed us to compare the amount of scar tissue development among the different groups.

### 2.7. Contractile Force Measurements

The isometric strength of ankle dorsiflexor muscles, which include the* tibialis anterior* muscle, was assessed in the animals from the d35* Control* and treatment groups as well as in another 4 animals, which were measured before and after myectomy (*Control no lesion* and* Control d0*, resp.), using a specially designed dynamometer that allows measuring the torque generated by rat's ankle muscles. With animals under deep anaesthesia with urethane (20% vol/vol, 6 mL/kg b.w.), the sciatic and the common fibular nerves were exposed at the level of the distal third of the thigh. The nerves were mobilized and released from the surrounding tissues, and the tibial nerve was cut to allow electrical stimulating of only the muscles supplied by the common fibular nerve. A cuff bipolar electrode, composed by two thin conductive wires held by a patch of a flexible tube, was placed encircling the common fibular nerve to electrically stimulate the nerve.

Animals were laid down in dorsal recumbent position on top of a heated pad, with hip and knee flexed at 90 degrees, and the foot strapped to the footplate with the ankle joint axis aligned with that of the footplate. The torque generated by ankle dorsiflexors was measured by a load cell (Futek, LSB200, sensitivity 2 mV/V, Irvine, CA, USA) connected to the front end of the footplate.

The torque generated by tetanic isometric contractions of the ankle dorsiflexor muscles was measured every 10° of ankle position between 120° and 40° of ankle's range of motion (0° the foot aligned with tibia). Muscle contractions were elicited by trains of supramaximal electrical pulses 0.5 seconds in duration, (stimulus frequency 150 Hz; current 4 mA) delivered to the common fibular nerve.

Force signals were recorded at 1 KHz sampling rate, analog-to-digital converted by a 12-bit card (Plux Wireless Biosignals, Arruda dos Vinhos, Portugal) and stored in a computer hard-disk for later processing. Ankle dorsiflexor muscles peak torque was obtained from the maximum torque value of each contraction. In few animals, force measurements were performed before and soon following the injury of the tibialis anterior muscle. Force measurements in the other animals were carried out at day 35 (d35).

### 2.8. Immunohistochemistry (IHC) Analysis

**Figure 3:**
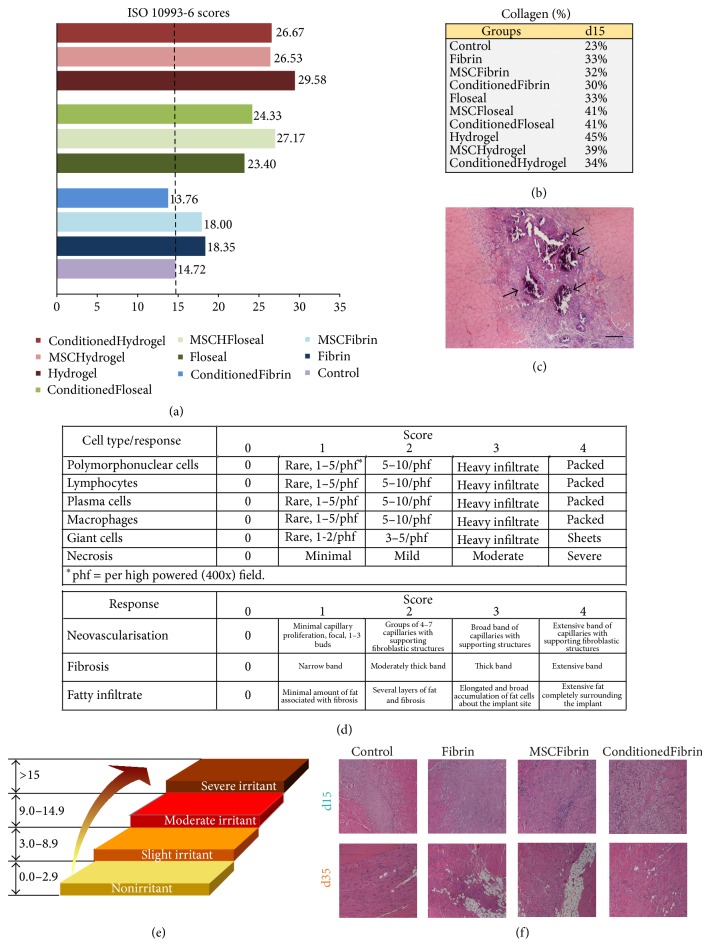
ISO 10993-6 scoring for the groups tested at d15. The* Control* group obtained a score of 14.72. The tested implants can be considered as slight, moderate, severe, and nonirritant depending on the score obtained in comparison to the* Control* group (a). Collagen fractions for the groups tested at d15 (b). Histological appearance (H&E staining) of a* tibialis anterior* (TA) muscle with exuberant calcification (arrows) after Floseal implantation (magnification: 40x). Scale bar: 50 *μ*m (c). Scoring system used for biological evaluation of degradable materials taking into account inflammation, neovascularization, fibrosis, and fatty infiltrate-ISO 10993-6 Annex E (d). International Standard classification of the biomaterials attributed by the results obtained through the previous scoring system: the average subtotal inflammation score is multiplied by 2 and added to the average neovascularization, fibrosis, and fatty infiltrate subtotal score. The final score for the tested biomaterial is the value obtained above (or below) the Control score (e). Histological appearance (H&E staining) from Control, Fibrin, MSCFibrin, and* ConditionedFibrin* TA muscles at d15 and d35 for the assessment of inflammatory status (f).

The most important event during muscle reconstruction is the activation of satellite cells. They are characterized by the expression of transcription factor Pax7 which is a paired box protein [9]. Through IHC, we tried to localize these cells within the injury area of the muscles where fibrin glue was used as a vehicle (*Fibrin*,* MSCFibrin*, and* ConditionedFibrin* at both d15 and d35). As* Control*, the same analysis in noninjured* tibialis anterior* muscles was performed (Figure 3).

Sections were deparaffinised and hydrated and antigen unmasking technique was performed using a pressure cooker with 10 mmol/L sodium citrate buffer, pH 6,0 for 3 min. Slides were cooled for 10 min at room temperature and rinsed twice in triphosphate buffered saline (TBS) for 5 min. Endogenous peroxidase was blocked with hydrogen peroxide 3% in methanol for 10 min. Immunohistochemistry was performed using the avidin-biotin-peroxidase complex (ABC) method, employing the monoclonal antisera Pax7 (chick Pax7 a.a. 352–523, Developmental Studies Hybridoma Bank, Iowa University) diluted 1 : 50. Sections were rinsed with TBS after each step of the procedure. Colour was developed for up to 7 min at room temperature with a freshly prepared solution of DAB and sections were then lightly counterstained with haematoxylin, dehydrated, and mounted. Negative controls were performed by replacing the primary antibody with TBS.

The Pax7 labelling index was defined as the percentage of Pax7 positive nuclei, which was determined by counting at least 1000 nuclei in the selected fields, at high power field magnification (×400). Multiple fields were necessary to obtain 1000 nuclei for each lesion.

### 2.9. Statistical Analysis

When relevant, data is presented as mean and standard deviation (SD). Statistical analysis was made by two-way ANOVA (time × group) for collagen area fraction data and one-way ANOVA for muscle strength data, followed by Tukey's pairwise comparisons. Force data before and immediately after* tibialis anterior* myectomy were compared by paired-sampled* t*-test. Statistical significance was accepted at *P* < 0.05. For the viability analysis, all data was presented as mean ± SEM, where *N* is the number of cells where the [Ca^2+^]_*i*_ was measured by the epifluorescence technique. For each experimental condition, 25 HMSCs were analyzed (*N* = 25).

## 3. Results

### 3.1. HMSCs Characterization and Cell Viability

The MSCs exhibited a mesenchymal-like shape with a flat and polygonal morphology. During expansion, the cells became long spindle-shaped and colonized the whole culturing surface (Figure 1). The phenotype of the HMSCs expanded for the* in vivo* testing was confirmed in our laboratory by flow cytometry in order to certify that the HMSCs followed the International Society for Cellular Therapy (ISCT) criteria [26]. As expected for MSC-type stem cells, flow cytometry analyses showed that over 96% of the cells in the population were consistently positive for the cell surface markers CD44, CD73, CD90, and CD105 and less than 2% positive for CD14, CD19, CD31, CD34, CD45, and HLA-DR.

The karyotype analysis to the HMSCs cell line derived from Human Wharton's jelly demonstrated that this cell line has not neoplastic characteristics and is stable during the cell culture procedures in terms of number and structure of the somatic and sexual chromosomes. The transplanted HMSCs also presented normal morphology and immunocytochemistry markers for MSCs.

Results obtained from epifluorescence technique referred to measurements from undifferentiated HMSCs, which correspond to [Ca^2+^]_*i*_ from cells that did not begin the apoptosis process. The [Ca^2+^]_*i*_ was 42.9 ± 4.5 (*N* = 25), 44.2 ± 3.5 (*N* = 25), and 50.1 ± 3.9 (*N* = 25) for HMSCs cultured in the presence of Floseal, fibrin matrix from Tisseellyo, and spherical hydrogel after 7 days of culture, respectively. The undifferentiated HMSCs cultured in the presence of the three tested vehicles reached confluence and exhibited a normal star-like shape with a flat morphology in culture. According to these results, it is reasonable to conclude that the three vehicles are viable substrates for undifferentiated HMSCs culture and survival and may be used in the preclinical trials.

### 3.2. Histological Evaluation of Local Effects

**Figure 4:**
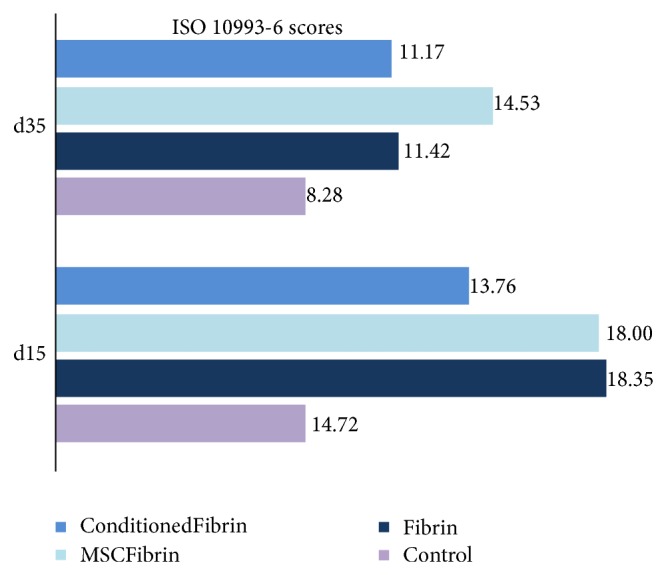
ISO 10993-6 scoring for the* Control* and treatment groups (after choosing fibrin glue—Tisseellyo as the preferred vehicle) tested at d15 and d35.

The histological analysis and ISO 10993-6 scoring proved that fibrin glue (score of 3.6 points above* Control*) is less reactive as a vehicle compared to the* Floseal* group (score of 8.68 points above* Control*) and* Hydrogel* (score of 10.94 points above* Control*) (Figure 3(a)). Overall, at d15 the only group considered as nonirritant was the* ConditionFibrin* group;* Fibrin*,* MSCFibrin*, and* Floseal* were considered slight irritant;* MSCFloseal*,* ConditionedFloseal*, and all the hydrogel groups were considered moderate irritants. Although the inflammatory cell population was considered to be more abundant in the* Hydrogel* group in terms of the ISO scoring, the hemostatic matrix (Floseal) produced an exuberant calcification (Figure 3(c)). In the groups treated with the CM instead of HMSCs, a blunted inflammatory response seemed to occur (an average of 2.31 points higher in the HMSCs groups in comparison to the CM treated groups). The effect of HMSCs in reducing inflammation was not noteworthy; at d15 and at d35, the number of inflammatory cells was higher compared to the* Control* groups. It should also be noticed that the score obtained at d35 for the* Fibrin* group was slightly superior to the group where CM was added to the fibrin glue (Figure 4).

### 3.3. Collagen Area and Collagen Content Fraction

**Figure 5:**
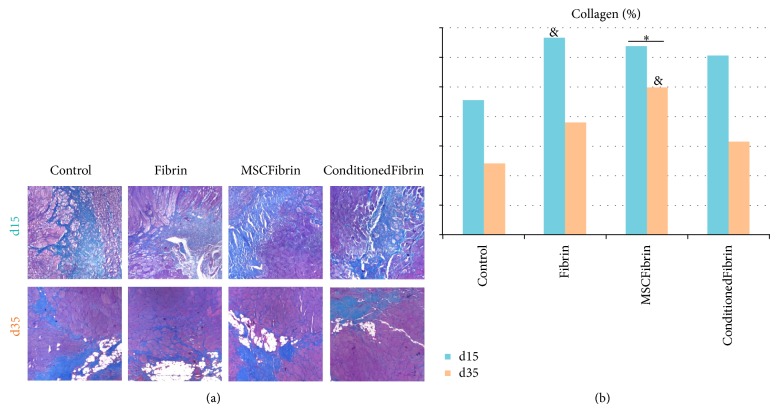
Histological analysis of* tibialis anterior* (TA) muscles from the* Control* and treatment groups (after choosing fibrin glue—Tisseellyo as the preferred vehicle) evaluated at d15 and d35: Masson's Thricrome/eosin-stained sections appearance (magnification: 40x). Scale bar: 50 *μ*m (a). Histomorphometric quantification of mean collagen fraction (b). ∗Significantly different from* Control* (d15 and d35 data pooled together; *P* < 0.05); ^&^significantly different from* Control* at the same time point (*P* < 0.05).

Two-way ANOVA demonstrated significant differences between groups (*Control*,* Fibrin*,* MSCFibrin*, and* ConditionedFibrin*) (*P* = 0.015) and between d15 and d35 (*P* < 0.001) in the fraction area occupied by collagen (fibrosis) but no group per time interaction effect (*P* = 0.636). Pooling d15 and d35 animals together, significantly higher collagen area fraction was observed in the* MSCFibrin* group compared to the* Control* group (*P* = 0.013). No other differences existed in collagen area fraction between the groups when considering the two time points together. Although no interaction was found between the different treatments and the time, we analyzed the data of collagen area and collagen content fraction at each time point with one-way ANOVA. At d15, again, greater collagen area fraction was found in the* Fibrin* group compared with the* Control* group (*P* = 0.045). At this time point, collagen area fraction was similar in the other groups. At d35, collagen area fraction was higher in the* MSCFibrin* group than in the* Control* group (*P* = 0.031) without further differences between the remaining groups (Figure 5).

### 3.4. Muscle Force

**Figure 6:**
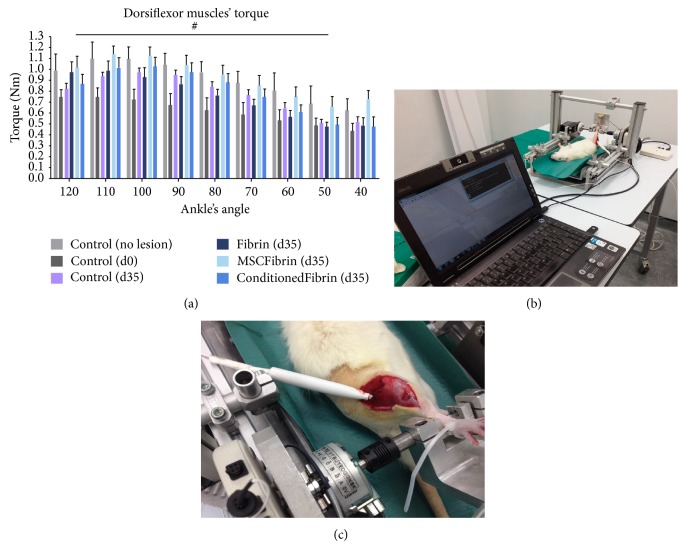
Dorsiflexor muscles' torque at different ankle joint angles for the rats from the* Control* and treatment groups (after choosing fibrin glue—Tisseellyo as the preferred vehicle) (a–c).

Ankle's dorsiflexor torque in intact animals displayed a characteristic pattern of variation across the joint's range of motion, with mean isometric peak torque occurring at 110 degrees of ankle flexion, then steadily declining with plantarflexion (Figure 6). The myectomy injury of the* tibialis anterior* muscle caused a reduction of approximately one-third in isometric peak torque generated by ankle dorsiflexor muscles (1.097 ± 0.309, 0.746 ± 0.172 Nm, pre- and postmyectomy, resp.). This decrease in isometric peak torque as a result of acute* tibialis anterior* muscle injury was apparent at every ankle angle tested (ANOVA: *P* = 0.006), although the difference to preinjury values could not reach statistical significance at the more extended ankle angle of 40°. In the* Control* group at d35, dorsiflexor isometric peak torque recovered to values similar to those of intact animals (1.097 ± 0.309 and 0.977 ± 0.092 Nm,* Control* animals premyectomy and* Control d35* animals d35 postmyectomy; ANOVA: *P* = 0.203, nonsignificant). Also, at d35 dorsiflexor isometric peak torque was similar in every muscle-injure group, irrespectively of the treatment (ANOVA: *P* = 0.181, nonsignificant).

### 3.5. Immunohistochemistry (IHC) Analysis

**Figure 7:**
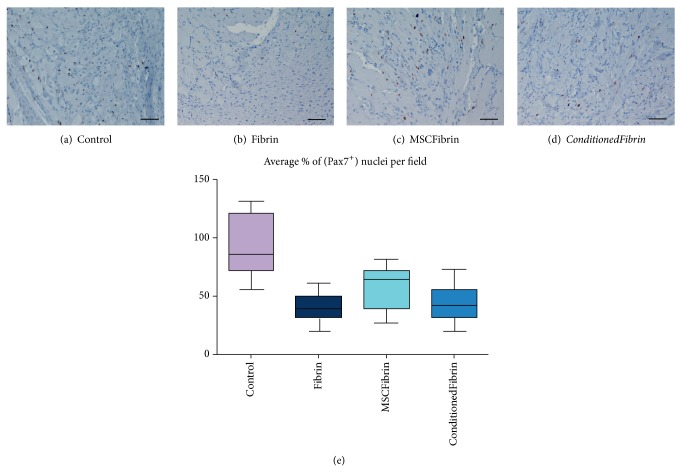
IHC sections of* tibialis anterior* (TA) muscles from the* Control* and treatment groups (after choosing fibrin glue—Tisseellyo as the preferred vehicle) with anti-human Pax7 antibody evaluated at d15 postmyectomy (magnification: 200x) (a–d). The Pax7^+^ labeling index was defined by the average number of Pax7^+^ positive nuclei which was determined by counting 5 selected fields at high power field magnification (×400) in each sample (e). Scale bar: 50 *μ*m.

**Figure 8:**
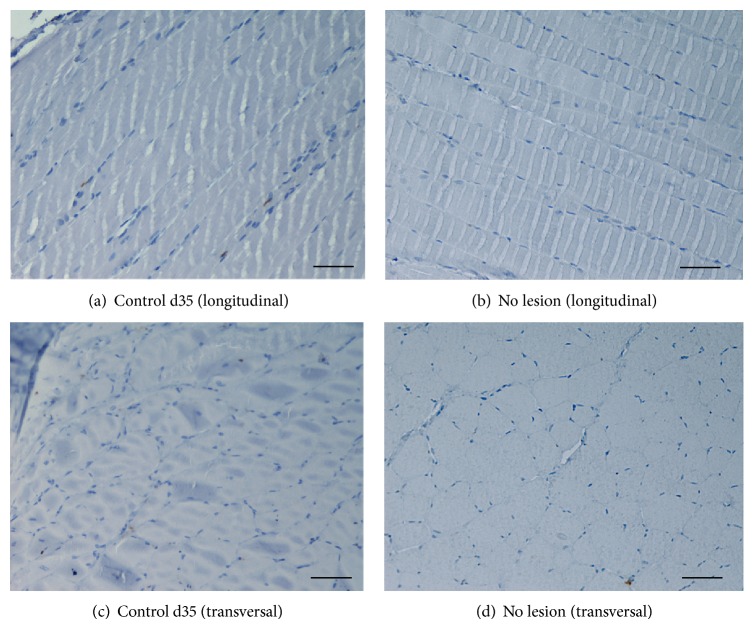
IHC sections from* tibialis anterior* (TA) muscles: the presence of Pax7^+^ nuclei in the* Control* (a and c) group (evaluated at d35 postmyectomy) was similar to the noninjured TA muscles (b and d) (magnification: 200x). Scale bar: 50 *μ*m.

At d15 postmyectomy, the number of Pax7^+^ nuclei from the* Control* group was clearly higher than all the treatment groups. However, the differences between the treatment groups were apparently slight (Figure 7). Nevertheless, the qualitative assessment of these IHC samples allowed us to observe that the degree of inflammation and also the amount of fibrosis were somehow related to the presence of Pax7^+^ nuclei. In fact, the muscles that were treated only with fibrin seemed to have an increased inflammatory cell population and amount of fibrosis with few Pax7^+^ nuclei. In the muscles treated with fibrin and HMSCs (*MSCFibrin* group), a more active inflammatory status and a slightly increased amount of fibrosis were not followed by a decrease in the number of Pax7^+^ nuclei when compared to the* Conditioned* group. It also must be stated that, for the muscles collected at d35 postmyectomy, the number of Pax7^+^ positive nuclei was insignificant in all the groups (*Control*,* Fibrin*,* MSCFibrin*, and* ConditionedFibrin*). It was also possible do observe that, in terms of Pax7^+^ cells, the noninjured* tibialis anterior* muscles were similar to the* Control* muscles at d35, which were also both insignificant (Figure 8).

## 4. Discussion

Skeletal muscle injuries are common in humans, particularly in athletes, and it is important to develop new methods to improve muscle regeneration. Skeletal muscle has good regenerative ability, but the extent of muscle injury might prevent complete regeneration, especially in terms of functional recovery. Severe lesions, like those originated by trauma associated with loss of healthy muscular tissue and development of fibrous tissue scar and irreversible muscular atrophy after long-term peripheral nervous injuries, are examples of those situations where regeneration is limited. An alternative approach for the restoration of the damaged skeletal muscular tissue are the methods that characterize the Regenerative Medicine which is the transplantation of stem cells that limit the fibrosis and the atrophy of the involved muscle masses and even imply the myocytes regeneration and local revascularization, associated with biomaterials or vehicles for those cell therapies. The* in vivo* application of HMSCs isolated from Wharton's jelly of the umbilical cord and the CM where the HMSCs were cultured and expanded, associated with different biocompatible vehicles to induce muscle regeneration in a rat anterior tibial myectomy model, was tested in the present study. It was known at the starting point of our study that, in other disease models characterized by inflammation sites, HMSCs transiently home and niche to inflammatory sites, remaining viable in a xenotransplated rat for up to approximately 2 weeks in normal mice/rats, increasing their permanence in inflammation sites in diseased models, without detectable homing to other organs. For example, the biodistribution of placental HMSCs, stably infected with a lentiviral construct expressing the luciferase gene, was performed in both immune-competent and immune-compromised NOD/SCID and Balb/c mice. When 1 × 10^6^ placental HMSCs were administrated, the biodistribution pattern showed that the cells persisted only at the injection site and did not distribute to other organs. As a matter of fact, placental HMSCs retained consistently high levels of luciferase expression,* in vitro*, for up to 3 weeks [31]. So, the HMSCs can modulate locally the inflammatory process improving the regeneration process of the tissue. Interestingly, these cells, which are major histocompatibility complex (MHC) class II negative, are able to both evade and modulate the immune system [32–34], making them an attractive cell source for MSC-based therapies including the skeletal muscle diseases and injuries. In addition, these cells represent a noncontroversial source of primitive mesenchymal progenitor cells that can be isolated after birth, cryogenically stored, thawed, and expanded for therapeutic uses [35]. Mesenchymal stem cells and new bioengineering approaches can create a supply of myogenic stem cells or implants applicable for acute and/or chronic muscle disorders. HMSCs can influence the myofiber regeneration and scar tissue formation processes mainly by their paracrine effect through a range of biomolecules synthesized by these cells, more than their direct differentiation into functional tissue. This recent paradigm has suggested that the biomolecules synthesized by stem cells may be as important, if not more so, as the differentiation of the cells in eliciting functional tissue repair [26]. This evidence is compatible with the results presented here, which suggest that the CM obtained from the* in vitro* culture and expansion of these cells could be an alternative therapeutic option compared to the* in vivo* transplantation of these stem cells, as it can benefit from the local tissue response to the secreted molecules without the difficulties and complications associated with the engraftment of the allotransplanted or xenotransplanted cells. Theoretically, these concerns should not be a relevant issue since MSCs present low immunogenicity and high immunosuppressive properties due to a decreased or even absence of human leucocyte antigen (HLA) class II expression [36]. However, the results described in this study reflect a slight long-term negative effect of the HMSCs, resulting in a better outcome in the groups treated with the CM both in the inflammatory status (ISO scoring) and in the collagen content for the samples evaluated near complete regeneration (d35 postinjury). In fact, at this time point and pooling d15 and d35 together, the collagen fractions from the* Control* groups were significantly different from the* MSCFibrin* groups but not from the* ConditionedFibrin* groups. With the exception of the* ConditionedFibrin* group (that expressed the best result for the ISO scoring at d15), all of the other treatment groups were considered inferior when compared to the* Control* groups (myectomy lesion only). It should be noticed that only in the latter was no vehicle implanted, which is probably the key point for this outcome.

The uses of constructs for tissue engineering (TE) and Regenerative Medicine are promising innovative therapies that can address several clinical situations, where the traumatic lesions of the skeletal muscle are included. These constructs are often a combination of cells, scaffolds, and biological factors. Although there are only a few commercial products currently in the market for cell/drug delivery, probably because each type of cell requires its own specific encapsulating microenvironment with cell-specific material properties and spatially controlled bioactive features, a vast amount of research is being performed worldwide on all aspects of tissue engineering/Regenerative Medicine exploring polymer materials. To implant cells into defective skeletal muscles, there are two main techniques. The cellular system may be directly injected into the scaffold which is localized in the injury site. It can also be performed by preadding the cells to the scaffold via injection or coculture (in most of the cellular systems, cells are allowed to form a monolayer) and then the biomaterial with the cellular system is implanted in the injured muscle. In case of multiple sites of injury, the systemic administration of cells capable of reaching damaged tissues would be an interesting alternative.

In this study, the association of the HMSCs with biocompatible vehicles that could slowly interact with the host tissue was selected, bearing in mind the absorption rate of these biomaterials. This strategy permits a gradual release of the cells/growth factors, rather than injecting them directly in the core or surroundings of the lesion, which would certainly predispose to a significant loss of these products immediately after delivery. The histological analysis and ISO 10993-6 scoring indicates that fibrin glue (Tisseellyo) is less reactive as a vehicle compared to Floseal or the hydrogel tested. Fibrin alone or in combination with other materials has been used as a biological scaffold for stem cells [37]. From our data, fibrin glue would probably be the most appropriate vehicle for implanting HMSCs or the CM in the developed myectomy injury model. It must be stated that taking the ISO 10993-6 general considerations, for degradable/resorbable biomaterials such as the ones used in this trial, the test period for histological evaluation should be related to the estimated degradation time of the vehicles. Only after a complete degradation and adsorption of the scaffolds, a steady state could be obtained. However, this standard also recommends the evaluation of the tissue response at intermediate degradation stages, in order to analyse the local adverse responses to the residual implant and its degradation products [28]. In our study, d15 and d35 as the two time points in the healing process were elected, for histological evaluation. These two periods permitted us to gather these recommendations to data obtained by our preliminary studies in order to focus our attention on both an incomplete and a complete stage of rat skeletal muscle regeneration [5]. The fact that in the second time point (d35) the number of Pax7^+^ nuclei was clearly not relevant is compatible with the indication that all these muscles revealed an almost complete regeneration.

A variety of experimental models that compromise skeletal muscle function or even destroy this tissue have been described throughout the years. Injections of myotoxic agents, mechanical crush, ischemia, denervation, or muscular dystrophies are commonly induced in different animal models in order to establish new approaches for the treatment of skeletal muscle diseases. However, severe lesions like traumatic injuries, associated, for example, with the handling of heavy machinery that occasionally result in labour accidents or the more frequently observed road accidents, are usually a challenge for orthopedic and cosmetic surgery and might me hard to mimic in the models described above. Tissue engineering and Regenerative Medicine are expected to contribute in a better recovery of these patients. Lacerating a muscle [6–8] or complementing it with the removal (myectomy) of a portion of muscular tissue [2, 4] has currently been elected as a more accurate model to disrupt muscular regeneration in a more emphasized manner. In a previous developed histological qualitative assessment, the myectomy model proved to be the most appropriate for a comprehensive and standard evaluation of the rat skeletal muscular regeneration [25], especially considering new biomaterials and cell therapies as described in here.

The basic concept of tissue engineering consists in the seeding of cells in a biodegradable scaffold impregnated or not with growth factors and/or cytokines [38]. The use of extraembryonic tissues as stem cell reservoirs for tissue engineering offers many advantages over both embryonic and adult stem cell sources. Most significantly, the comparatively large volume of extraembryonic tissues and the easiness of physical manipulation theoretically increase the number of stem cells that can be isolated [39–42].

The MSCs are characterized by several important and distinct properties, such as the following: (a) being plastic adherent; (b) having specific surface protein expression, staining positively for markers of the mesenchymal lineage like CD10, CD13, CD29, CD44, CD90, and CD105 and negatively for markers of the hematopoietic lineage; and (c) having a tri-lineage differentiation capacity of the isolated MSCs. These are the criteria that were defined by the International Society for Cellular Therapy (ISCT), which are followed by the HMSCs used in this preclinical trial [43]. The production of growth factors and cytokines is an important capacity of these cells. The MSCs from Wharton's jelly of the umbilical cord, as it was previously demonstrated concerning the UCX cellular product from ECBIO, SA, grown in aggregates that better mimic tissue environment, have produced a secretome rich in trophic factors, such as HGF, TGF-*β*, G-CSF, VEGF-A, FGF-2, KGF, and IL-6 that promote wound healing reactions, as demonstrated both* in vitro* by vasculogenesis, mitogenic, and chemotactic assays and* in vivo* using a chemotaxis assay where MSCs were shown to recruit the surrounding bone marrow MSCs known to be directly involved in tissue regeneration [44]. This important capacity was also studied by other research groups. Jackson and collaborators, in 2012, referred that, in cases where the inflammatory phase is prolonged, the proinflammatory mediators, such as myofibroblasts and fibrocytes, will promote the generation of a nonfunctional tissue that will result in the formation of a scar [45]. MSCs secrete a variety of cytokines and growth factors like hepatocyte growth factor (HGF), IL-10, or adrenomedullin that may, among other effects, suppress the local immune system, enhance angiogenesis (they produce basic FGF and VEGF-A), or inhibit scar formation (by the antifibrotic properties of the previously mentioned factors) [45–48]. By attenuating the function of B-cells and natural killer cells, the MSCs would likely reduce the profibrotic responses that can occur coincidently with prolonged inflammation during wound healing [45, 49]. These suggestions may explain the results obtained for the collagen type I quantification of the treated groups at d15 postmyectomy, in which, apart from the groups with Floseal as a vehicle, a less evident scar tissue was present in the muscles treated with HMSCs or their CM (Figure 3(c)). In fact, Floseal alone revealed an inferior collagen fraction (compared to* MSCFloseal* and* ConditionedFloseal* groups) but this line of evidence was clearly a result of the exuberant calcification produced by this vehicle, which was even more evident in the muscles that where not treated with MSCs nor with the CM (Figures 3(b)-3(c)). This line of evidence was also stated in the ISO 10993-6 scoring, in which Floseal alone also resulted in a better scoring in terms of inflammatory status (Figure 3(a)). Nevertheless, one should consider that the observed necrosis and calcification may not be properly assessed by these analyses, which could in fact misrepresent our judgment about the applicability of this vehicle. For that reason, the hemostatic matrix (Floseal) was considered inappropriate for being used as MSCs' vehicle.

Once the MSCs enter the inflammatory environment, their immunomodulatory phenotype becomes activated by IFN*γ*, TNF*α*, and IL-1*β*. There is also evidence that MSCs are capable of ameliorating the acute immune response to injury by the previously mentioned suppression of B-cells and natural killer cells [45, 49]. Surprisingly in this study, the effect of HMSCs in reducing inflammation was not remarkable at d15 and at d35; the number of inflammatory cells was even higher compared to the* Control* group. In terms of this histological evaluation performed at d35 postmyectomy, in animals where the myectomy injury was filled with fibrin glue associated with the cellular system, it is interesting to observe that the HMSCs apparently have a long-term negative effect in terms of chronic inflammation (Figure 4). At d15,* Conditioned* groups also presented better or similar ISO scores to* MSC* groups but the increased number of Pax7^+^ cells in the* MSCFibrin* group at d15 (Figure 7(e)) may suggest that this effect has not a direct association with the quality and degree of regeneration since our semi-quantitative evaluation (ISO scoring) was based only on the inflammation features of the damaged muscles.

Grabowska et al. [12] previously demonstrated that, under the* in vivo* muscle microenvironment conditions, HMSCs are only sporadically included into newly reconstructed myotubes, but their presence had a beneficial effect on the injured muscle regeneration. The lack of incorporation of MSCs in host tissues has engendered controversy about their use and this inability to target these cells to tissues of interest with high efficiency and engraftment is considered a significant barrier to the effective implementation of MSC therapy [50]. However, it has been proposed that MSCs modulate the host environment by indirect mechanisms. They appear to provide some paracrine trophic effect by the delivery of complex signals (that may, in part, be mediated by modulators of Wnt signaling) to a target tissue rather than being involved in tissue restoration as direct participants, through their incorporation in the host tissues [51, 52]. It has been confirmed that grafted MSCs do not remain in the site of injury when transplanted nor do they translocate to other regions, suggesting that their role is largely limited to signaling that initiates the recruitment of endogenous cell to the affected areas [52]. For example, the ability of Wnt signaling to induce myogenic differentiation and to promote proliferation and migration in Sprague-Dawley rats' MSCs has also been proven [53]. Having this into consideration in this study, the CM collected from the HMSCs expansion culture was tested independently, since the CM might include the secreted proteins which play a central role in the previously mentioned* in vivo* signaling process. The HMSCs' local application might not have the required therapeutic efficiency, since the cells may migrate to different body regions and tissues or might not survive and produce the appropriate trophic factors. These pitfalls are overcome by the local application of CM associated with a biocompatible vehicle.

## 5. Conclusions

The groups treated with the different vehicles and HMSCs obtained the expected results in terms of muscle regeneration, both in the histological and in the functional assessments. Functionally, it was concluded that, at d35 dorsiflexor, isometric peak torque recovered to values similar to intact muscles regardless the treatment option. We are confident in considering that the use of cellular products (particularly CM) should not be discarded as a viable clinical option for the treatment of skeletal muscle injuries. Searching for an optimal vehicle with minimal influence in terms of biological incorporation associated with MSCs from Wharton's jelly or with CM enriched by growth factors and cytokines produced by this cellular system might be the key point in the future of skeletal muscle tissue engineering and the use of cellular therapies in muscular defects.
